# Excess healthcare costs of mental disorders in children, adolescents and young adults in the Basque population registry adjusted for socioeconomic status and sex

**DOI:** 10.1186/s12962-023-00428-w

**Published:** 2023-03-01

**Authors:** Igor Larrañaga, Oliver Ibarrondo, Lorea Mar-Barrutia, Myriam Soto-Gordoa, Javier Mar

**Affiliations:** 1 Research Unit, Osakidetza Basque Health Service, Debagoiena Integrated Health Organisation, Avenida Navarra 16, 20500 Arrasate-Mondragón, Gipuzkoa Spain; 2 Kronikgune Institute for Health Services Research, Barakaldo, Spain; 3 Biodonostia Health Research Institute, Donostia-San Sebastián, Spain; 4 Osakidetza Basque Health Service, Araba University Hospital, Vitoria-Gasteiz, Spain; 5 Faculty of Engineering, Mondragon Unibertsitatea, Arrasate-Mondragón, Gipuzkoa Spain

**Keywords:** Excess costs, Mental disorders, Socioeconomic status, Gender, Young adults

## Abstract

**Background:**

Mental illnesses account for a considerable proportion of the global burden of disease. Economic evaluation of public policies and interventions aimed at mental health is crucial to inform decisions and improve the provision of healthcare services, but experts highlight that nowadays the cost implications of mental illness are not properly quantified. The objective was to measure the costs of excess use of all healthcare services by 1- to 30-year-olds in the Basque population as a function of whether or not they had a mental disorder diagnosis.

**Methods:**

A real-world data study was used to identify diagnoses of mental disorders and to measure resource use in the Basque Health Service Registry in 2018. Diagnoses were aggregated into eight diagnostic clusters: anxiety, attention deficit hyperactivity disorder, conduct disorders, mood disorders, substance use, psychosis and personality disorders, eating disorders, and self-harm. We calculated the costs incurred by each individual by multiplying the resource use by the unit costs. Annual costs for each cluster were compared with those for individuals with no diagnosed mental disorders through entropy balancing and two-part models which adjusted for socioeconomic status (SES).

**Results:**

Of the 609,381 individuals included, 96,671 (15.9%) had ≥ 1 mental disorder diagnosis. The annual cost per person was two-fold higher in the group diagnosed with mental disorders (€699.7) than that with no diagnoses (€274.6). For all clusters, annual excess costs associated with mental disorders were significant. The adjustment also evidenced a social gradient in healthcare costs, individuals with lower SES consuming more resources than those with medium and higher SES across all clusters. Nonetheless, the effect of being diagnosed with a mental disorder had a greater impact on the mean and excess costs than SES.

**Conclusions:**

Results were consistent in showing that young people with mental disorders place a greater burden on healthcare services. Excess costs were higher for severe mental disorders like self-harm and psychoses, and lower SES individuals incurred, overall, more than twice the costs per person with no diagnoses. A socioeconomic gradient was notable, excess costs being higher in low SES individuals than those with a high-to-medium SES. Differences by sex were also statistically significant but their sizes were smaller than those related to SES.

**Supplementary Information:**

The online version contains supplementary material available at 10.1186/s12962-023-00428-w.

## Background

Mental illnesses account for a considerable proportion (10%) of the global burden of disease [1, 2]. The literature suggests that preventive interventions at early age are key to tackle adverse conditions experienced during childhood and adolescence and contribute to better levels of health in adulthood [3]. Therefore, economic evaluation of public policies and interventions aimed to reduce the burden is crucial to inform decisions about what is the best use of the limited resources available in order to maximize the health benefits [4]. Nonetheless, the cost of mental disorders is a poorly understood driver of decision-making about which interventions should be implemented in mental health [5, 6]. More economic evaluations in the field of mental illness have started to be conducted [7–10], but their limited use in decision-making contrasts with the importance placed on this type of research in the incorporation of preventive treatments and interventions in cancer and cardiovascular diseases [11–13]. Moreover, experts highlight that the cost implications are not adequately measured and large evidence gaps still exist regarding the economic case for mental health care [14], including inequalities by gender and socioeconomic status (SES) [6].

In this context, data are needed on excess healthcare costs associated with mental disorders for various purposes, such as conducting economic evaluations and measuring the burden [15]. To measure disease-specific burden, average costs should be disaggregated by the type of mental disorder and compared with those for similar populations without such disorders [15–18]. That is, there is a need to know not only the average cost but also the incremental cost in relation to the population without mental health diagnoses [17, 18]. In this sense, if a new intervention can modify the costs or benefits in health associated to a given mental disorder, the burden of the new scenario proposed by the intervention can be obtained and compared with the current scenario in order to guide decision-making [5, 6].

Currently, the relatively few data available are based on surveys collecting self-reported data from samples of patients with diagnoses of specific mental disorders [8, 17–19]. In this field, there is a lack of real-world data (RWD) studies, despite such research having been recognized by experts as a key source of information for understanding disease-specific resource use [20]. As RWD provide information on individual resource use for an entire population, analysis of these types of data makes it possible to measure population costs [21]. In turn, having population data disaggregated at the individual level, accurate unit costs can be provided to be used to estimate the economic burden of health disorders and to carry out subsequent cost analysis of interventions [3]. RWD also help explore differences in health, since groups that make greater use of health resources are generally those that have poorer health status [22, 23]. The monitoring of health disparities allows us to measure progress toward achieving health equity and social justice [24]. As people diagnosed with a mental disorder tend to use health services more than the general population, health service use may reveal trends in disparities in mental health [25, 26].

Besides mental disorders, inequities in resource use can also be associated with SES and mental disorders in populations that are strongly determined by socioeconomic characteristics [23, 27, 28]. Moreover, due to a greater vulnerability to environmental stress in the early stages of life, social determinants have more impact on children, adolescents and young adults [29, 30]. Therefore, knowing the joint impact of mental disorders and SES on healthcare costs would help to assess both, their burden and their relationship with social determinants [31].

Given all this, the objective of this study was to measure the excess use of healthcare resources and healthcare costs of people between 1 and 30 years of age in the Basque population, adjusted for SES and sex, as a function of whether or not they are diagnosed with some type of mental disorder.

## Methods

A retrospective observational study was conducted to identify diagnoses of mental disorders and to measure resource use based on data from the Basque Health Service. The Basque Country is an industrialized northern region of Spain with a population of 2.2 million. In Spain, powers for managing health services are decentralised to the regions, and the health system recognises a universal right to healthcare under a Beveridge model. The Basque Health Service provides comprehensive healthcare to the entire Basque population. The protocol of the study was approved by the Clinical Research Ethics Committee of the Basque Country (number PI2019078).

The Basque Health Service registry contains information on all psychiatric and somatic inpatient and outpatient encounters (admissions and consultations), primary care contacts and emergency room visits. Diagnoses are recorded using codes from the ninth and tenth revisions of the International Classifications of Diseases (ICD-9 and ICD-10). In the study, the definition of lifetime prevalence of Kessler et al. was applied, who estimated it as the proportion of respondents who had ever been diagnosed with a given disorder up to their age at interview [32]. Based on this prevalence-based approach, we calculated, first, the resources used (primary care, mental health centres, hospitals and pharmacy) and, second, the corresponding direct costs. By merging diagnoses and resource use in the population registry, we obtained individual data for the whole population disaggregated by clusters of mental disorders.

The study population was all individuals who, as of 31 December 2018, were between 1 and 30 years old and registered with the Basque Health Service. Among this population, patients who had been diagnosed with any mental disorder at any point in their lifetime were identified by checking all lifetime episodes of primary care and hospital care. Diagnoses were aggregated into eight diagnostic clusters: anxiety (anxiety + acute stress reactions + adaptation reactions), attention deficit hyperactivity disorder (ADHD), conduct disorders, mood disorders (depression + bipolar disorder), substance use, psychosis and personality disorders, eating disorders, and self-harm. In addition, as patients from private practice seek drug reimbursement through the public system, we searched for individuals who had any relevant chronic prescriptions through Anatomical Therapeutic Chemical (ATC) codes for antidepressants (N06A group) or antipsychotics (N05A group) in individuals without a mental disorder diagnosis in the public health service records to include them in the clusters of mood disorders and psychosis respectively. In the identification process, we used the ICD-9, ICD-10 and ATC classification system codes (listed in Additional file 1: Table S1).

The variables included in the study were: age, sex and income level based on drug co-payment, and diagnosis cluster. In addition, all the resource use of the target population was extracted for the year 2018. That way, the resource use profile of the general population was estimated. Data collected in primary care included all contacts with nurses and general practitioners at healthcare centres, at home or by telephone. For hospital care, we took into account all contacts with outpatient clinics, as well as with emergency and inpatient services. All the drugs prescribed to individuals were also considered. The information about the unit costs of different healthcare resources for 2018 in euros (EUR, €) was obtained from the accounting system of the Basque Health Service (Additional file 1: Table S2) and included all types of healthcare resources [salaries, diagnosis costs (Lab, Rx), equipment, investments, infrastructure (heating, electricity, cleaning services, etc.) and pharmacy].

To assess SES, we considered drug co-payment categories which are established based on household income (Additional file 1: Table S3). The contribution levels for the co-payment of medicines in the Spanish Health System were established in 2012 based on three criteria: income, age and degree of illness. Children, adolescents and young adults were assigned the most disadvantaged SES level (low SES) if the head of their household was exempt from co-payment or was retired, the most advantaged SES level (high SES) if the head of the household had an annual income from paid work equal or higher than €18,000, and otherwise, to a third category (medium SES), for heads of household with annual incomes from paid work lower than €18,000 [27].

We calculated the costs incurred by each individual multiplying the resource use by the unit costs. As individual data were available, the cost per patient was disaggregated into primary care, hospital care and pharmacy costs for each diagnostic cluster.

### Statistical analysis

In the initial step, univariate statistical analysis was performed to compare the sociodemographic features of individuals with and without mental disorders. Fisher’s exact test was used for categorical variables with two categories and expected values less than or equal to 5, and otherwise a chi-square test. In the case of age, since it is a continuous variable with a normal distribution, the comparison of means was carried out using Student’s t test.

In a second step, each diagnostic cluster was compared with the population with no diagnosed mental disorders to measure the excess cost using statistical models. In total, 10 independent statistical models were created: one for each diagnostic cluster, one for having two diagnoses or more, and one for having any mental disorder. Before developing the models, the data were pre-processed. When using nonrandomized studies to estimate costs, it must be taken into account that selection is influenced by individual characteristics. Since initial characteristics were likely to be different in the groups with and without mental disorders, they were balanced to ensure that they were comparable in terms of initial characteristics (age group, sex and SES) and independent of background characteristics. For that purpose, an entropy balancing technique was used to adjust the covariate distribution of the group with no diagnosed mental disorders by reweighting. This technique is based on a maximum entropy reweighting scheme and allows the pre-processing of data in observational studies with binary variables of interest [18, 33]. The technique reweighted the data from no diagnosed mental disorders units to match a set of moments that was computed from the data from the group with mental disorders. Hence, the covariate distribution obtained was more similar to that in the group with mental disorders. In that way, the covariate distributions in the reweighted data satisfied the balance conditions specified by the research team and the resulting weights were used to carry out an analysis comparing the two groups, where confounding factors between them were removed. As ten different models were built to analyse excess costs, covariate distribution adjustment using entropy balancing was also performed once per model.

To measure the excess costs, it would not have been appropriate to use ordinary least squares regression models [34], since the costs did not follow a normal distribution and a substantial number of individuals had zero costs. Therefore, to obtain the excess costs for each mental health cluster and adjusted for the selected characteristics, regression analysis was performed using two-part models [18, 34, 35]. In the first part, the adjusted probability, p(x), that the cost was higher than zero was fitted with a logit regression model. In the second part, generalized linear models with a log link function and gamma distribution were used to calculate the mean cost values of the population with costs greater than zero. An advantage of two-part models is that their results are easy to interpret since they estimate the magnitude of the differences between groups (in our case, in costs) and not only if the compared means differ. As the estimated costs depend on the combination of the different covariates, they produce the average costs for each cluster and the adjusted excess costs.

All the statistical analyses were carried out using R (version 3.3.2) and Stata (version 14) statistical programs with a significance level of 95%. Specifically, the initial univariate statistical analysis was performed with R, which is free, while the entropy balancing and two-part models were performed with Stata, to take advantage of dedicated packages available, namely, *ebalance* and *twopm*, respectively [36, 37].

## Results

The total population in the age range between 1 and 30 years included in the Basque Health Service database contained 609,381 individuals, of which 96,671 (15.9%) had been diagnosed with at least one mental disorder at some point in their lifetime (Table 1). The SES distribution confirmed the social gradient in mental disorders, the prevalence rising from low SES to medium and high SES groups (also in Table 1). The lifetime prevalence of mental disorders and the use of resources in the population studied disaggregated by diagnostic cluster are presented in Table 2 and Additional file 1: Table S4 disaggregated by cluster. They show that the gradient according to SES is a pattern repeated in all the diagnostic clusters. Anxiety was the most prevalent type of mental disorder, diagnoses in this cluster being recorded in 6.6% of the population under 30 years. Individuals with more than one diagnosis appear in various clusters. Among the entire population with mental disorders, 20% had two or more diagnoses. Notably, Table 2 shows the greater use of healthcare resources by people with diagnosed mental disorders. Their rate of admission to psychiatric wards was higher (0.7%) than that in people with no diagnoses (0.0%). But notably their rate of admission to general wards was also nearly two-fold higher (5.4% versus 2.8% in the population with no diagnosed mental disorders). Individuals with mental disorders also incurred noticeably higher annual drug prescription costs (€115.6 versus €33.7).

**Table 1:** Characteristics of the study population between 1 and 30 years of age as of 31 December 2018 with and without a diagnosed mental disorder (individuals 1–30 years of age, Basque Health Service registry 31 December 2018)

		Study population		Diagnosed mental disorder				p-value
				No		Yes		
		N	%	N	%	N	%	
Patients	Total	609,381		512,710	84.1	96,671	15.9	
Age (years)	Mean	15.57		14.69		20.23		< 0.001^a^
	0–12	246,275	40.4	231,079	93.8	15,196	6.2	< 0.001^b^
	13–18	123,109	20.2	100,582	81.7	22,527	18.3	
	18–24	114,710	18.8	87,918	76.6	26,792	23.4	
	25–30	125,287	20.6	93,131	74.3	32,156	25.7	
Sex	Female	296,556	48.7	251,825	84.9	44,731	15.1	< 0.001^c^
	Male	312,825	51.3	260,885	83.4	51,940	16.6	
Socioeconomic status	Low	47,416	7.8	36,945	77.9	10,471	22.1	< 0.001^b^
	Medium	312,135	51.2	254,306	81.5	57,829	18.5	
	High	249,830	41.0	221,459	88.6	28,371	11.4	

^a^Calculated using Student’s t test for continuous variables

^b^Calculated using chi-square tests

^c^Calculated using Fisher’s exact test

**Table 2:** Percentage of use of each resource over 1 year by the prevalence of mental disorders in the Basque population under 30 years

	Population size	Primary care (%)	Outpatient services		Emergency services (%)	Inpatient services		Intensive care (%)
			Total (%)	Psychiatry (%)		Total (%)	Psychiatry (%)	
General population	609,381	63.0	34.8	3.3	26.4	3.2	0.1	0.2
Population with no diagnosed mental disorders	512,710 (84.1%)	60.5	31.8	1.0	25.3	2.8	0.0	0.2
Population with ≥ 1 diagnosed mental disorder	96,671 (15.9%)	76.5	50.6	15.3	32.5	5.4	0.7	0.2
Substance use	19,507 (3.2%)	80.9	46.7	11.7	38.2	9.4	2.0	0.4
Anxiety	40,523 (6.6%)	83.3	52.5	14.9	36.3	6.5	1.0	0.3
Mood disorders	8,613 (1.4%)	81.0	69.7	42.0	38.1	11.7	6.1	0.5
Psychosis and personality disorders	5.745 (0.9%)	77.7	69.1	46.0	41.4	13.5	8.1	0.7
Attention deficit hyperactivity disorder	16,986 (2.8%)	71.6	51.1	18.4	27.9	3.9	0.6	0.2
Conduct disorders	26,415 (4.3%)	71.6	53.6	20.9	33.6	5.0	1.2	0.2
Eating disorders	4,629 (0.8%)	77.4	55.1	18.6	33.3	6.6	1.8	0.3
Self-harm	664 (0.1%)	85.5	72.7	52.3	59.0	27.1	20.3	2.1
2 or more diagnoses	19,567 (3.2%)	83.5	61.9	29.6	41.3	9.6	3.3	0.4

The annual costs per person disaggregated by diagnostic group and cost component are listed in Table 3 and Additional file 1: Table S5. Hospital costs represented three quarters of the total cost. As patients with more than one diagnosis may be included in various clusters, the overall mean does not match the weighted average of the clusters. The total healthcare cost per person in the diagnosed group (€699.7) was more than twice that in the group with no diagnoses (€274.6). The clusters that consumed the most resources were self-harm, with mean costs of €4543.7, followed by psychosis and personality disorders with costs of €2359.8 and mood disorders with costs of €1874.7.

**Table 3:** Mean direct healthcare costs per person in € (2018) disaggregated by diagnostic group and cost component

	Total costs	Primary care costs		Non-psychiatric hospital care costs		Psychiatric hospital care costs		Drug prescription costs	
General population	342.0	44.8	13.1%	220.1	64.4%	30.4	8.9%	46.7	13.6%
Population with no diagnosed mental disorder	274.6	37.1	13.5%	199.9	72.8%	3.9	1.4%	33.7	12.3%
Population with ≥ 1 diagnosed mental disorder	699.7	85.7	12.2%	326.9	46.7%	171.5	24.5%	115.6	16.5%
Substance use	1012.7	107.4	10.6%	420.4	41.5%	363.8	35.9%	121.2	12.0%
Anxiety	813.0	116.4	14.3%	377.5	46.4%	204.9	25.2%	114.1	14.0%
Mood disorders	1874.7	115.2	6.1%	487.8	26.0%	1,157.2	61.7%	114.6	6.1%
Psychosis and personality disorders	2,359.8	102.4	4.3%	549.5	23.3%	1,591.0	67.4%	116.8	4.9%
Attention deficit hyperactivity disorder	619.5	63.8	10.3%	255.1	41.2%	183.3	29.6%	117.3	18.9%
Conduct disorders	778.3	64.4	8.3%	305.7	39.3%	294.1	37.8%	114.2	14.7%
Eating disorders	1,070.8	87.0	8.1%	424.6	39.7%	443.3	41.4%	115.9	10.8%
Self-harm	4,543.7	156.4	3.4%	931.4	20.5%	3,338.8	73.5%	117.1	2.6%
2 or more diagnoses	1,335.2	121.1	9.1%	465.7	34.9%	633.8	47.5%	114.5	8.6%

For all the models developed, the balance achieved by entropy balancing (Additional file 1: Tables S6–15), the two-part models with their parameters (Additional file 1: Tables S16–25) and the results on mean and excess cost of the combined statistical analysis are provided in the supplementary material (Additional file 1: Tables S26–35). To summarise our results here, we present the mean and excess costs per patient by diagnostic cluster and disaggregated by SES in Table 4, sex in Table 5 and age-group in Table 6. For all clusters, annual excess costs in the groups of patients with mental disorders were more than double those in the groups with no diagnosed mental disorders. Tables 5, 6 also show the differences in adjusted costs by sex and age group. For all clusters, annual excess costs were higher in women than in men. Disaggregation by age group did not render a fully consistent pattern, but in general, younger age groups incurred lower excess costs.

**Table 4:** Mean and excess cost per patient in € (2018) of direct healthcare costs disaggregated by socioeconomic status and diagnostic group

		Mean cost (€)^a^		Excess cost (€)	p-value^a^
		With no mental disorders (N = 512,710)	With mental disorder(s)		
Any mental disorder (N = 96,671)	High	242	610	368	< 0.001
	Medium	270	684	414	< 0.001
	Low	404	1007	603	< 0.001
Substance use (N = 19,507)	High	215	762	547	< 0.001
	Medium	258	924	666	< 0.001
	Low	556	1952	1395	< 0.001
Anxiety (N = 40,523)	High	251	696	445	< 0.001
	Medium	280	785	505	< 0.001
	Low	448	1235	787	< 0.001
Mood disorders (N = 8613)	High	251	1557	1306	< 0.001
	Medium	273	1700	1428	< 0.001
	Low	466	2855	2389	< 0.001
Psychosis and personality disorders (N = 5.745)	High	237	1875	1638	< 0.001
	Medium	262	2097	1835	< 0.001
	Low	434	3411	2977	< 0.001
Attention deficit hyperactivity disorder (N = 16,986)	High	228	568	339	< 0.001
	Medium	249	617	368	< 0.001
	Low	328	803	475	< 0.001
Conduct disorder (N = 26,415)	High	246	687	441	< 0.001
	Medium	265	744	479	< 0.001
	Low	378	1047	668	< 0.001
Eating disorder (N = 4629)	High	260	894	634	< 0.001
	Medium	295	1018	723	< 0.001
	Low	486	1642	1156	< 0.001
Self-harm (N = 664)	High	339	4692	4353	< 0.001
	Medium	258	3486	3228	< 0.001
	Low	514	6816	6302	< 0.001
2 or more diagnoses (N = 19,567)	High	245	1142	897	< 0.001
	Medium	262	1232	970	< 0.001
	Low	445	2055	1610	< 0.001

^a^Calculated using two-part models and groups were adjusted by age group, sex and SES using entropy balancing

**Table 5:** Mean and excess cost per patient in € (2018) of direct healthcare costs disaggregated by sex and diagnostic group

		Mean cost (€)^a^		Excess cost (€)	p-value^a^
		With no mental disorders (N = 512,710)	With mental disorder(s)		
Any mental disorder (N = 96,671)	Female	310	770	460	< 0.001
	Male	246	633	387	< 0.001
Substance use (N = 19,507)	Female	323	1118	795	< 0.001
	Male	247	904	657	< 0.001
Anxiety (N = 40,523)	Female	321	880	560	< 0.001
	Male	245	704	459	< 0.001
Mood disorders (N = 8613)	Female	317	1923	1606	< 0.001
	Male	277	1763	1485	< 0.001
Psychosis and personality disorders (N = 5.745)	Female	334	2584	2250	< 0.001
	Male	268	2167	1899	< 0.001
Attention deficit hyperactivity disorder (N = 16,986)	Female	277	677	400	< 0.001
	Male	240	598	358	< 0.001
Conduct disorder (N = 26,415)	Female	292	808	515	< 0.001
	Male	263	741	478	< 0.001
Eating disorder (N = 4629)	Female	326	1111	785	< 0.001
	Male	245	859	614	< 0.001
Self-harm (N = 664)	Female	353	4727	4374	< 0.001
	Male	265	3636	3371	< 0.001
2 or more diagnoses (N = 19,567)	Female	317	1446	1129	< 0.001
	Male	255	1226	971	< 0.001

^a^Calculated using two-part models and groups were adjusted by age group, sex and SES using entropy balancing

**Table 6:** Excess cost per patient in € (2018) of direct healthcare costs disaggregated by age group and diagnostic group

		Mean cost (€)		Excess cost (€)	p-value^a^
		Without mental disorder (N = 512,710)	With mental disorder		
Any mental disorder (N = 96,671)	1–12	287	702	415	< 0.001
	13–18	269	675	406	< 0.001
	19–24	255	648	393	< 0.001
	25–30	293	751	457	< 0.001
Substance use (N = 19,507)	1–12	364	1.222	858	< 0.001
	13–18	326	1.130	804	< 0.001
	19–24	270	955	685	< 0.001
	25–30	280	1.004	724	< 0.001
Anxiety (N = 40,523)	1–12	276	745	469	< 0.001
	13–18	306	848	543	< 0.001
	19–24	269	747	478	< 0.001
	25–30	301	847	546	< 0.001
Mood disorders (N = 8613)	1–12	251	1.492	1.240	< 0.001
	13–18	332	2.036	1.705	< 0.001
	19–24	277	1.726	1.450	< 0.001
	25–30	308	1.940	1.632	< 0.001
Psychosis and personality disorders (N = 5.745)	1–12	247	1.869	1.623	< 0.001
	13–18	296	2.324	2.028	< 0.001
	19–24	289	2.306	2.017	< 0.001
	25–30	306	2.472	2.166	< 0.001
Attention deficit hyperactivity disorder (N = 16,986)	1–12	281	671	390	< 0.001
	13–18	253	623	370	< 0.001
	19–24	227	571	345	< 0.001
	25–30	257	659	403	< 0.001
Conduct disorder (N = 26,415)	1–12	262	716	454	< 0.001
	13–18	270	755	485	< 0.001
	19–24	267	757	490	< 0.001
	25–30	332	947	616	< 0.001
Eating disorder (N = 4629)	1–12	272	918	646	< 0.001
	13–18	331	1.137	806	< 0.001
	19–24	267	920	653	< 0.001
	25–30	337	1.164	827	< 0.001
Self-harm (N = 664)	1–12	163	2.058	1.895	< 0.001
	13–18	378	5.040	4.661	< 0.001
	19–24	255	3.429	3.175	< 0.001
	25–30	366	5.027	4.661	< 0.001
2 or more diagnoses (N = 19,567)	1–12	281	1.253	972	< 0.001
	13–18	303	1.397	1.094	< 0.001
	19–24	262	1.228	966	< 0.001
	25–30	293	1.388	1.096	< 0.001

^a^Calculated using two-part models and groups were adjusted by age group, sex and SES using entropy balancing

## Discussion

To our knowledge, this is the first study showing individual excess costs of persons with mental diagnoses and adjusted for SES and sex covering a general population of 609,381 individuals younger than 30 years old. Children, adolescents and young adults diagnosed with mental disorders used health services more and this implied a high excess cost, the annual cost per diagnosed person being, overall, more than twice the cost per person with no diagnoses. A socioeconomic gradient was notable, excess costs being higher in individuals with low SES than those with high-to-medium SES. The low SES category (7.8%) grouped the adolescents and young people in households with no income with those whose health cardholder was on benefits and exempt from payment or retired regardless of their income (i.e., with an income lower or higher than €18,000). The rationale for this can be seen in Additional file 1: Table S3 which shows that adolescents and young people depending on a retired cardholder had a higher prevalence of mental disorders (24.2% and 20.5%) and consistent with SES relying not only on income but also on family structure. Differences by sex were also statistically significant but their sizes were smaller than those related to SES. Our cluster-disaggregated prevalence results for 18-year-olds are consistent with those described in Denmark in a population registry base study [38].

The healthcare costs were comprehensive as they included hospital care, primary care and pharmacy. Roughly three quarters of the costs per patient were hospital-related costs, which included those for emergency services and specialized outpatient clinics as well as hospital ward admissions. In another registry-based study, Christensen et al. estimated the total healthcare cost of all persons living in Denmark with a diagnosis of mental disorder [39]. When comparing with their mean annual healthcare costs, as would be expected for a country with lower salaries, our annual costs were in a lower range, but the ratio between the annual healthcare costs in diagnosed and non-diagnosed individuals was roughly three in both studies. When analysing the results on annual excess healthcare costs, they also found that schizophrenia and drug use disorders incurred the highest ones. However, the different age range of the two populations hampered the comparison with our results as we limited our study to individuals from 1 to 30 years and the somatic burden is much higher in older cohorts [40].

On the other hand, the mean total costs were within the range of the real per capita health spending by age group in Spain estimated by top-down methods and the estimated annual costs were also quite similar in both studies [41]. The excess costs were important in all three cost components, differences in hospital costs being greater in absolute terms, but the relative difference in pharmacy was also considerable. Drug prescription costs were 3.4 times higher in the group with mental diagnoses, revealing the use of psychoactive drugs in all age groups under 30 years.

Two diagnosis clusters generated the highest costs per individual, self-harm with costs of €4543.7 and psychosis and personality disorders with costs of €2359.8. After statistical adjustment using the two-part models, they continued to be the clusters with the highest average and highest excess costs. When disaggregating by SES, the social gradient is reflected in the statistical models and the top figure of €6,302 was obtained for the self-harm cluster in the low SES group. The differences by SES are striking in all the clusters and especially between, on the one hand, low SES, and, on the other, medium and high SES categories (Table 2 and Additional file 1: Table S4) [42]. At this point it is important to remark that, as long as universal coverage is provided, any citizen has guaranteed access to health services. Nevertheless, it is possible that differences found may undervalue the whole reality when looking to the literature. Findings that children from low SES families respond more strongly to cost sharing policies such as co-payments [43], acting as a barrier when seeking healthcare assistance, suggest that there can be an underestimation in this group. Moreover, social and cultural factors like stigma and negative perceptions surrounding mental illness can also influence the use of healthcare system, especially conditioning the access of the most vulnerable groups [44]. Therefore, actual differences between SES groups could even increase.

When looking over the effect of sex, the total spending by females is greater than by males in coherence with the literature [45, 46]. In the same way, the analyses revealed that, in terms of excess cost, women’s also had higher numbers in each diagnostic cluster. The higher total and excess costs found in females can be explained because women tend to use more the healthcare services in general [45, 46]. There are differences in cluster prevalence by sex, but they did not bias the excess cost calculation thanks to the adjustment achieved with the two-part models.

It is noteworthy that individuals with mental disorders incurred higher costs not only for mental healthcare but also for somatic healthcare. A similar pattern of use has been found elsewhere among under 18-year-olds diagnosed with a mental disorder [47]. In adults, the higher resource use has been partially attributed to chronic comorbidities [48], but specific explanations are required for young people with very few chronic physical conditions. As suggested by the literature, a possible justification can be that the presence of a mental disorder was associated with an increased risk of subsequent medical conditions [49]. Different studies also indicate that parental coping with a mental illness is related to the mental health of their children [50–52], as well as with the increase in their healthcare services use [53, 54]. However, it must be taken into account that people with mental health disorders are a heterogeneous group with different health and social needs, where the drivers of their higher resource use are likely to be multifactorial [48]. It must also be considered that healthcare use and cost estimates in adolescents and young adults may be underestimated, as long as practitioners can be reluctant to diagnose certain disorders, especially more severe ones, until the patient reaches an older age [55, 56]. Therefore, initiatives should be developed to improve early recognition and mental health support for young people, seeking both to improve their care and potentially reduce inappropriate care and costs [57].

The availability of data on the excess costs of mental disorders opens an opportunity for undertaking studies on the effectiveness and cost-effectiveness of preventive interventions in adolescents [3]. In particular, reducing the incidence of self-harm, psychoses and personality disorders and mood disorders should be considered a public health priority, because these disorders are associated with disability, and also have serious economic consequences. The relevance of these findings is underlined by the effect of the coronavirus disease 2019 pandemic on the mental health of adolescents and has major implications for prevention planning [58, 59]. Preventive interventions for self-harm and suicide must be included in the guidelines to safeguard the mental health of adolescents and young adults affected by the pandemic and the measures restricting social mobility, with a focus on measures to mitigate anxiety, depression, and stigma, among other conditions.

## Limitations and strengths

Our study was carried out from the perspective of the health system and therefore our data lack the weight of other categories such as social, judicial and educational costs. We acknowledge that a fully comprehensive approach to estimating the burden of mental disorders must incorporate a societal perspective by covering all cost categories assessed in top-down cost-of-illness studies such as data on crime, accidents and social care [17, 60]. Moreover, informal costs due to caregivers’ time should be accepted as part of the economic burden of mental disorders but so far these key components are not recorded in registries [61]. Our figures for excess costs would have been even higher if those cost categories had been measured [18]. Further, while the economic impact of informal care is important, so is the suffering and loss of quality of life of siblings who endure the care of children, adolescents and young adults with mental disorders [61]. Wittenberg et al. described this situation highlighting “health as a family affair” [61].

Another limitation of the study was the lack of validation of the diagnoses. As in other observational studies, the cohort effect may bias the results [38]. Our dataset is based on the integration of information on all the diagnoses of individuals recorded in the electronic health record of the public health service in their contacts with primary, hospital inpatient, emergency and outpatient care. This approach yielded consistent results in the diagnosis of dementia in various European countries [62]. In the Basque Country, nearly universal health coverage is provided, but in the age range studied, 20% of the population also have private insurance. This cost component is absent in our database and therefore its size was not considered. The percentage of high-income individuals with double coverage (public and private) is greater in high SES people, and they may opt to use private rather than public providers, and hence, the differences by SES may be biased [63]. The lack of adjustment for comorbidities was also a potential limitation. Nonetheless, in these early stages of life, social determinants have a greater impact on health than physical chronic conditions [29]. Finally, another limitation of the study was the definition of the different clusters of mental disorders. Our approach to classifying mental disorders roughly followed the categories defined by Dalsgaard et al. for the same purpose also using ICD-10 codes and a population registry [38]. In contrast, self-reported symptoms in surveys are converted into codes from the successive versions of the Diagnostic and Statistical Manual of Mental Disorders (DSM-IV), to estimate prevalence indicators [32].

Besides its limitations, our study has important strengths. As the data were derived from a population-based registry covering the whole population, it provides a recent and comprehensive estimate of the direct medical costs for a population of more than half a million individuals under 30 years of age. Furthermore, the registry contained the contacts with healthcare providers in all the public settings in the Basque Country. A similar approach has been successfully applied in Denmark to estimate incidence rates of mental disorders [38, 64]. The joint use of Basque administrative and clinical databases allowed us to obtain population-based cost estimates across the entire healthcare system avoiding the selection bias associated with small samples from psychiatric settings. Another strength is the availability of all contacts to measure the resource use at the patient level directly, instead of relying on patient-reported healthcare use or top-down approaches [7, 16, 17, 65].

The current availability of information from electronic health records enables the undertaking of observational studies based on RWD that allow the measurement of actual resource use and costs. Nevertheless, the high external validity of these types of study may be weakened given their non-random design, where the baseline characteristics of the groups to be compared can differ due to selection bias. To overcome this issue, pre-processing techniques like entropy balancing or propensity score matching are crucial to adjust the covariate distribution of the control group by the reweighting or discarding of units [33, 66, 67]. Such techniques make the distribution more similar to the one in the comparison group. In this case, entropy balancing was used to carry out this task. In contrast to other pre-processing methods, this technique tackles the adjustment problem backwards and estimates the set of weights that satisfies the balance constraints that involve the first, second and higher moments of the covariate distributions as well as interactions. Because of that, a high degree of covariate balance can be obtained. Moreover, since entropy balancing weights show smooth variation across units, its appeal lies in its capacity to optimize the balance in the covariate distribution while retaining the maximum amount of information. Finally, compared to other techniques like propensity score matching, it can be faster computationally speaking, it being possible to obtain the weights within a few seconds even in large databases.

## Conclusions

This study provides estimates of the excess economic costs of mental disorders for the first time in the Spanish population between 1 and 30 years of age based on a general population registry. Results are consistent in showing that young people with mental disorders place a greater burden on healthcare providers compared to population without mental disorders, and that the costs are especially high for severe mental disorders like self-harm and psychoses. Additionally, the results on excess healthcare costs obtained may facilitate future economic evaluations of interventions targeting adolescents and young adults, supporting decision-making in order to improve the provision of mental healthcare services.

## Supplementary Information

**Additional file 1: **Healthcare Costs of Mental Disorders in Children, Adolescents and Young Adults in the Basque Population Registry Adjusted for Socioeconomic Status and Sex. Additional file 1 contains information about the ICD and ATC codes used to define each mental disorder category, the unit costs used, the distribution of prevalence of mental disorders by co-payment categories, the cost per patient of direct healthcare costs disaggregated by age and diagnostic group, the covariate balance achieved by entropy balancing, the two-part model parameters for each mental disorder category, and the mean and excess cost per patient of direct healthcare costs for each mental disorder category disaggregated by sex, age group and SES.

## Data Availability

Data were provided by the Basque Health Service. Our data sharing agreement stipulates that they cannot be shared with any third party.

## Abbreviations

RWD: Real-world data
SES: Socioeconomic status
ADHD: Attention deficit hyperactivity disorder
ATC: Anatomical therapeutic chemical
ICD: International classifications of diseases
