# Supplementary material for: Excess healthcare costs of mental disorders in children, adolescents and young adults in the Basque population registry adjusted for socioeconomic status and sex
Source: Cost Eff Resour Alloc. 2023 Mar 1;21:18. doi: 10.1186/s12962-023-00428-w (PMC9975849; doi:10.1186/s12962-023-00428-w)
Supplement: Supplementary file 1 — Additional file 1: Healthcare Costs of Mental Disorders in Children, Adolescents and Young Adults in the Basque Population Registry Adjusted for Socioeconomic Status and Sex. Additional file 1 contains information about the ICD and ATC codes used to define each mental disorder category, the unit costs used, the distribution of prevalence of mental disorders by co-payment categories, the cost per patient of direct healthcare costs disaggregated by age and diagnostic group, the covariate balance achieved by entropy balancing, the two-part model parameters for each mental disorder category, and the mean and excess cost per patient of direct healthcare costs for each mental disorder category disaggregated by sex, age group and SES. [file 12962_2023_428_MOESM1_ESM.pdf]

Excess Healthcare Costs of Mental Disorders in Children, Adolescents and Young Adults in the Basque Population Registry Adjusted for Socioeconomic Status and Sex. Supplementary material.

Table S1. ICD and ATC diagnostic codes used for each category of mental disorder.

	ICD-9	ICD-10	ATC
**Attention deficit hyperactivity disorder (ADHD)**	314.xx	F90.xx	
**Conduct disorders**	312.0x-312.2x, 312.4x-312.9x, 313.xx	F91.xx-F99.xx	
**Anxiety**	300.xx, 308.xx-309.xx	F40.xx-F49.xx	
**Depression**	296.xx-299.xx, 300.4, 311	F30.xx-F39.xx	Group N06A
**Substance abuse**	303.xx-305.xx	F10.xx-F19.xx	
**Psychosis and personality disorders**	291.xx-292.xx, 295.xx	F20.xx-F29.xx	Group N05A
	301.xx-302.xx, 312.3x	F60.xx-F69.xx	
**Eating disorders**	307.1x, 307.5x	F50.xx	
**Self-harm**	V62.84, E95x.xx	R45.851, T14.91, X71.xx-X83.xx	

Table S2. Unit costs of the healthcare resources obtained from the Basque Health Service for the year 2018.

Resource	Euros (€)
**PC nurse (centre)**	12.31
**PC nurse (telephone)**	6.16
**PC nurse (home)**	22.37
**General practitioner (centre)**	27.91
**General practitioner (telephone)**	13.95
**General practitioner (home)**	39.09
**Outpatient services (first)**	136.55
**Outpatient services (second)**	80.31
**A&E services**	172.68
**Hospitalisation (per day)**	486.02
**Home hospitalisation (per day)**	349.15

Table S3. Distribution of prevalence of mental disorders by co-payment categories.

Socioeconomic status	Co-payment category		N	%	Mental disorders (n)	Mental disorders (%)
	Code	Income				
Low	TSI 001 (unemployed)	No income	22,285	3.7%	4,655	20.9%
	TSI 002_01 (retired)	< €18,000	17,858	2.9%	4,322	24.2%
	TSI 002_02 (retired)	≥ €18,000	7,273	1.2%	1,494	20.5%
Medium	TSI 003 (active worker)	< €18,000	312,135	51.2%	57,829	18.5%
High	TSI 004 (active worker)	≥ €18,000	240,996	39.5%	27,606	11.5%
	TSI 005 (active worker)	≥ €100,000	6,952	1.1%	535	7.7%
	TSI 006 (active worker)	Special category (muface)	1,882	0.3%	230	12.2%
Total			609,381	100.0%	96,671	15.9%

Table S4. Prevalence of mental disorders in the Basque population under 30 years disaggregated by age, sex and socioeconomic status.

		**General population**		**Substance abuse**		**Anxiety**		**Mood disorders**		**Psychosis and personality disorders**		**ADHD**		**Conduct disorders**		**Eating disorders**		**Self-harm**	
		**N**	**%**	**N**	**%**	**N**	**%**	**N**	**%**	**N**	**%**	**N**	**%**	**N**	**%**				
**Patients**	**Total**	609,381	100.0%	19,507	3.2%	40,523	6.6%	8,613	1.4%	5,745	0.9%	16,986	2.8%	26,415	4.3%	4,629	0.8%	664	0.1%
**Age**	**0-12**	246,275	40.4%	10	0.0%	2,435	1.0%	1,148	0.5%	863	0.4%	2,896	1.2%	8,492	3.4%	955	0.4%	31	0.0%
	**13-18**	123,109	20.2%	1,427	1.2%	6,472	5.3%	1,473	1.2%	1,095	0.9%	6,829	5.5%	8,884	7.2%	1,118	0.9%	161	0.1%
	**18-24**	114,710	18.8%	6,548	5.7%	12,098	10.5%	2,444	2.1%	1,586	1.4%	5,405	4.7%	5,875	5.1%	1,316	1.1%	235	0.2%
	**25-30**	125,287	20.6%	11,522	9.2%	19,518	15.6%	3,548	2.8%	2,201	1.8%	1,856	1.5%	3,164	2.5%	1,240	1.0%	237	0.2%
**Sex**	**Women**	296,556	48.7%	8,364	2.8%	24,437	8.2%	3,970	1.3%	1,990	0.7%	4,421	1.5%	9,882	3.3%	3,261	1.1%	422	0.1%
	**Men**	312,825	51.3%	11,143	3.6%	16,086	5.1%	4,643	1.5%	3,755	1.2%	12,565	4.0%	16,533	5.3%	1,368	0.4%	242	0.1%
**Socioeconomic status**	**Low**	47,416	7.8%	1,916	4.0%	4,274	9.0%	1,284	2.7%	1,142	2.4%	1,880	4.0%	3,624	7.6%	465	1.0%	129	0.3%
	**Medium**	312,135	51.2%	14,152	4.5%	26,612	8.5%	5,230	1.7%	3,357	1.1%	8,642	2.8%	13,737	4.4%	2,604	0.8%	417	0.1%
	**High**	249,830	41.0%	3,439	1.4%	9,637	3.9%	2,099	0.8%	1,246	0.5%	6,464	2.6%	9,054	3.6%	1,560	0.6%	118	0.0%

Table S5. Mean direct healthcare costs per person in € (2018) disaggregated by age and diagnostic group.

**Total cost**	**Total**	**1-12 years**	**12-18 years**	**18-24 years**	**24-30 years**
General population	342.0	313.5	323.2	349.6	409.8
Population without mental health problems	274.6	295.9	237.7	249.3	285.6
Population with mental health problems	699.7	581.6	704.7	678.8	769.3
Substance abuse	1,012.7	1,207.5	1,266.3	1,014.2	980.4
Anxiety	813.0	565.3	967.8	774.1	816.8
Mood disorders	1,874.7	926.8	2,387.0	1,847.4	1,987.5
Psychosis and personality disorders	2,359.8	1,086.3	2,518.8	2,592.5	2,612.3
ADHD	619.5	595.1	646.3	575.4	687.1
Conduct disorders	778.3	565.6	818.3	847.7	1,108.3
Eating disorders	1,070.8	634.3	1,348.7	997.8	1,233.8
Self-harm	4,543.7	507.2	6,684.2	3,517.1	4,635.4
2 or more diagnoses	1,335.2	936.3	1,551.9	1,262.3	1,361.5
**Primary care costs**	**Total**	**1-12 years**	**12-18 years**	**18-24 years**	**24-30 years**
General population	44.8	19.2	48.6	66.6	71.6
Population without mental health problems	37.1	19.5	42.8	55.9	57.2
Population with mental health problems	85.7	15.9	74.6	101.4	113.2
Substance abuse	107.4	47.3	110.7	104.9	108.4
Anxiety	116.4	15.9	97.7	124.7	130.0
Mood disorders	115.2	15.9	91.5	129.2	147.5
Psychosis and personality disorders	102.4	14.7	100.2	114.2	129.4
ADHD	63.8	15.2	62.9	82.2	89.1
Conduct disorders	64.4	16.1	74.1	94.4	110.8
Eating disorders	87.0	17.1	83.1	110.8	118.9
Self-harm	156.4	11.0	147.7	153.4	184.4
2 or more diagnoses	121.1	16.0	103.4	128.8	142.7
**Hospital care costs**	**Total**	**1-12 years**	**12-18 years**	**18-24 years**	**24-30 years**
General population	250.5	256.5	227.4	229.0	281.5
Population without mental health problems	203.8	243.2	165.6	157.5	191.0
Population with mental health problems	498.4	457.9	503.0	463.4	543.5
Substance abuse	784.2	729.1	994.4	795.1	752.1
Anxiety	582.5	443.7	737.2	533.2	579.1
Mood disorders	1,644.9	796.5	2,162.9	1,604.4	1,732.4
Psychosis and personality disorders	2,140.6	965.4	2.276.2	2,356.0	2,378.6
ADHD	438.4	468.1	459.0	376.7	495.7
Conduct disorders	599.8	443.3	616.1	639.1	900.9
Eating disorders	867.9	507.8	1,151.1	770.2	993.6
Self-harm	4,270.2	406.1	6,411.5	3,256.1	4,326.4
2 or more diagnoses	1,099.5	813.6	1,306.6	1,019.7	1,115.3
**Drug prescription costs**	**Total**	**1-12 years**	**12-18 years**	**18-24 years**	**24-30 years**
General population	46.7	37.8	47.2	54.1	56.7
Population without mental health problems	33.7	33.2	29.3	35.8	37.5
Population with mental health problems	115.6	107.8	127.0	114.0	112.6
Substance abuse	121.2	431.0	161.2	114.2	119.9
Anxiety	114.1	105.7	132.9	116.3	107.7
Mood disorders	114.6	114.5	132.7	113.8	107.7
Psychosis and personality disorders	116.8	106.1	142.3	122.3	104.3
ADHD	117.3	111.8	124.4	116.5	102.3
Conduct disorders	114.2	106.1	128.2	114.1	96.6
Eating disorders	115.9	109.4	114.5	116.9	121.2
Self-harm	117.1	90.1	125.0	107.6	124.7
2 or more diagnoses	114.5	106.7	141.8	113.8	103.5

Table S6. The covariate balance achieved by entropy balancing in sex, age group and SES for the model of any mental disorder.

Before weighting	With any mental disorder			Without mental disorder		
	Mean	Variance	Skewness	Mean	Variance	Skewness
Sex[a]	0,5373	0,2486	-0,1496	0,5088	0,2499	-0,0354
Age group[b]	1,7850	1,1480	-0,3317	1,0840	1,3420	0,5373
SES[c]	0,8148	0,3675	0,1137	0,6401	0,3745	0,3973
**After weighting**	**With any mental disorder**			**Without mental disorder**		
	Mean	Variance	Skewness	Mean	Variance	Skewness
Sex[a]	0,5373	0,2486	-0,1496	0,5373	0,2486	-0,1495
Age group[b]	1,7850	1,1480	-0,3317	1,7850	1,1480	-0,3316
SES[c]	0,8148	0,3675	0,1137	0,8148	0,3675	0,1137

[a] Sex: 0=women; 1=men.

[b] Age group: 0=0-12 years; 1=13-18 years; 2=18-24 years; 3=25-30 years.

[c] SES: 0=high; 1=medium; 2=low.

* Comoments of the joint distribution of the covariates were also adjusted, but the balances achieved for the interactions are not shown in order to not overload the supplementary material with information.

Table S7. The covariate balance achieved by entropy balancing in sex, age group and SES for the model of substance use.

**Before weighting**	**With substance use**			**Without mental disorder**		
	Mean	Variance	Skewness	Mean	Variance	Skewness
Sex[a]	0,5712	0,2449	-0,2879	0,5088	0,2499	-0,0354
Age group[b]	2,5160	0,3991	-0,9628	1,0840	1,3420	0,5373
SES[c]	0,9219	0,2684	-0,1059	0,6401	0,3745	0,3973
**After weighting**	**With substance use**			**Without mental disorder**		
	Mean	Variance	Skewness	Mean	Variance	Skewness
Sex[a]	0,5712	0,2449	-0,2879	0,5712	0,2449	-0,2878
Age group[b]	2,5160	0,3991	-0,9628	2,5160	0,3991	-0,9629
SES[c]	0,9219	0,2684	-0,1059	0,9219	0,2684	-0,1060

[a] Sex: 0=women; 1=men.

[b] Age group: 0=0-12 years; 1=13-18 years; 2=18-24 years; 3=25-30 years.

[c] SES: 0=high; 1=medium; 2=low.

* Comoments of the joint distribution of the covariates were also adjusted, but the balances achieved for the interactions are not shown in order to not overload the supplementary material with information.

Table S8. The covariate balance achieved by entropy balancing in sex, age group and SES for the model of anxiety.

Before weighting	With anxiety			Without mental disorder		
	Mean	Variance	Skewness	Mean	Variance	Skewness
Sex[a]	0,3970	0,2394	0,4212	0,5088	0,2499	-0,0354
Age group[b]	2,2020	0,8410	-0,8765	1,0840	1,3420	0,5373
SES[c]	0,8677	0,3258	-0,0037	0,6401	0,3745	0,3973
**After weighting**	**With anxiety**			**Without mental disorder**		
	Mean	Variance	Skewness	Mean	Variance	Skewness
Sex[a]	0,3970	0,2394	0,4212	0,3970	0,2394	0,4212
Age group[b]	2,2020	0,8410	-0,8765	2,2020	0,8410	-0,8765
SES[c]	0,8677	0,3258	-0,0037	0,8677	0,3258	-0,0037

[a] Sex: 0=women; 1=men.

[b] Age group: 0=0-12 years; 1=13-18 years; 2=18-24 years; 3=25-30 years.

[c] SES: 0=high; 1=medium; 2=low.

* Comoments of the joint distribution of the covariates were also adjusted, but the balances achieved for the interactions are not shown in order to not overload the supplementary material with information.

Table S9. The covariate balance achieved by entropy balancing in sex, age group and SES for the model of mood disorders.

Before weighting	With mood disorders			Without mental disorder		
	Mean	Variance	Skewness	Mean	Variance	Skewness
Sex[a]	0,5391	0,2485	-0,1568	0,5088	0,2499	-0,0354
Age group[b]	1,9740	1,1160	-0,6277	1,0840	1,3420	0,5373
SES[c]	0,9054	0,3839	0,0638	0,6401	0,3745	0,3973
**After weighting**	**With mood disorders**			**Without mental disorder**		
	Mean	Variance	Skewness	Mean	Variance	Skewness
Sex[a]	0,5391	0,2485	-0,1568	0,5388	0,2485	-0,1556
Age group[b]	1,9740	1,1160	-0,6277	1,9730	1,1170	-0,6267
SES[c]	0,9054	0,3839	0,0638	0,9051	0,3839	0,0641

[a] Sex: 0=women; 1=men.

[b] Age group: 0=0-12 years; 1=13-18 years; 2=18-24 years; 3=25-30 years.

[c] SES: 0=high; 1=medium; 2=low.

* Comoments of the joint distribution of the covariates were also adjusted, but the balances achieved for the interactions are not shown in order to not overload the supplementary material with information.

Table S10. The covariate balance achieved by entropy balancing in sex, age group and SES for the model of psychosis and personality disorders.

**Before weighting**	**With psychosis and personality disorders**			**Without mental disorder**		
	Mean	Variance	Skewness	Mean	Variance	Skewness
Sex[a]	0,6536	0,2264	-0,6457	0,5088	0,2499	-0,0354
Age group[b]	1,8920	1,1630	-0,5035	1,0840	1,3420	0,5373
SES[c]	0,9819	0,4154	0,0167	0,6401	0,3745	0,3973
**After weighting**	**With psychosis and personality disorders**			**Without mental disorder**		
	Mean	Variance	Skewness	Mean	Variance	Skewness
Sex[a]	0,6536	0,2264	-0,6457	0,6536	0,2264	-0,6455
Age group[b]	1,8920	1,1630	-0,5035	1,8920	1,1630	-0,5034
SES[c]	0,9819	0,4154	0,0167	0,9819	0,4153	0,0167

[a] Sex: 0=women; 1=men.

[b] Age group: 0=0-12 years; 1=13-18 years; 2=18-24 years; 3=25-30 years.

[c] SES: 0=high; 1=medium; 2=low.

* Comoments of the joint distribution of the covariates were also adjusted, but the balances achieved for the interactions are not shown in order to not overload the supplementary material with information.

Table S11. The covariate balance achieved by entropy balancing in sex, age group and SES for the model of ADHD.

**Before weighting**	**With ADHD**			**Without mental disorder**		
	Mean	Variance	Skewness	Mean	Variance	Skewness
Sex[a]	0,7397	0,1925	-1,0930	0,5088	0,2499	-0,0354
Age group[b]	1,3660	0,7917	0,1461	1,0840	1,3420	0,5373
SES[c]	0,7301	0,4184	0,3271	0,6401	0,3745	0,3973
**After weighting**	**With ADHD**			**Without mental disorder**		
	Mean	Variance	Skewness	Mean	Variance	Skewness
Sex[a]	0,7397	0,1925	-1,0930	0,7396	0,1926	-1,0920
Age group[b]	1,3660	0,7917	0,1461	1,3660	0,7917	0,1462
SES[c]	0,7301	0,4184	0,3271	0,7301	0,4184	0,3271

[a] Sex: 0=women; 1=men.

[b] Age group: 0=0-12 years; 1=13-18 years; 2=18-24 years; 3=25-30 years.

[c] SES: 0=high; 1=medium; 2=low.

* Comoments of the joint distribution of the covariates were also adjusted, but the balances achieved for the interactions are not shown in order to not overload the supplementary material with information.

Table S12. The covariate balance achieved by entropy balancing in sex, age group and SES for the model of conduct disorders.

**Before weighting**	**With conduct disorders**			**Without mental disorder**		
	Mean	Variance	Skewness	Mean	Variance	Skewness
Sex[a]	0,6259	0,2342	-0,5203	0,5088	0,2499	-0,0354
Age group[b]	1,1400	1,0030	0,4314	1,0840	1,3420	0,5373
SES[c]	0,7944	0,4377	0,2523	0,6401	0,3745	0,3973
**After weighting**	**With conduct disorders**			**Without mental disorder**		
	Mean	Variance	Skewness	Mean	Variance	Skewness
Sex[a]	0,6259	0,2342	-0,5203	0,6256	0,2342	-0,5190
Age group[b]	1,1400	1,0030	0,4314	1,1400	1,0040	0,4317
SES[c]	0,7944	0,4377	0,2523	0,7941	0,4377	0,2528

[a] Sex: 0=women; 1=men.

[b] Age group: 0=0-12 years; 1=13-18 years; 2=18-24 years; 3=25-30 years.

[c] SES: 0=high; 1=medium; 2=low.

* Comoments of the joint distribution of the covariates were also adjusted, but the balances achieved for the interactions are not shown in order to not overload the supplementary material with information.

Table S13. The covariate balance achieved by entropy balancing in sex, age group and SES for the model of eating disorders.

**Before weighting**	**With eating disorders**			**Without mental disorder**		
	Mean	Variance	Skewness	Mean	Variance	Skewness
Sex[a]	0,2955	0,2082	0,8963	0,5088	0,2499	-0,0354
Age group[b]	1,6140	1,1860	-0,1494	1,0840	1,3420	0,5373
SES[c]	0,7634	0,3816	0,2012	0,6401	0,3745	0,3973
**After weighting**	**With eating disorders**			**Without mental disorder**		
	Mean	Variance	Skewness	Mean	Variance	Skewness
Sex[a]	0,2955	0,2082	0,8963	0,2958	0,2083	0,8946
Age group[b]	1,6140	1,1860	-0,1494	1,6120	1,1870	-0,1482
SES[c]	0,7634	0,3816	0,2012	0,7631	0,3815	0,2016

[a] Sex: 0=women; 1=men.

[b] Age group: 0=0-12 years; 1=13-18 years; 2=18-24 years; 3=25-30 years.

[c] SES: 0=high; 1=medium; 2=low.

* Comoments of the joint distribution of the covariates were also adjusted, but the balances achieved for the interactions are not shown in order to not overload the supplementary material with information.

Table S14. The covariate balance achieved by entropy balancing in sex, age group and SES for the model of self-harm.

Before weighting	With self-harm			Without mental disorder		
	Mean	Variance	Skewness	Mean	Variance	Skewness
Sex[a]	0,3645	0,2320	0,5633	0,5088	0,2499	-0,0354
Age group[b]	2,0210	0,7869	-0,4433	1,0840	1,3420	0,5373
SES[c]	1,0170	0,3723	-0,0084	0,6401	0,3745	0,3973
**After weighting**	**With self-harm**			**Without mental disorder**		
	Mean	Variance	Skewness	Mean	Variance	Skewness
Sex[a]	0,3645	0,2320	0,5633	0,3648	0,2317	0,5618
Age group[b]	2,0210	0,7869	-0,4433	2,0200	0,7864	-0,4426
SES[c]	1,0170	0,3723	-0,0084	1,0170	0,3710	-0,0084

[a] Sex: 0=women; 1=men.

[b] Age group: 0=0-12 years; 1=13-18 years; 2=18-24 years; 3=25-30 years.

[c] SES: 0=high; 1=medium; 2=low.

* Comoments of the joint distribution of the covariates were also adjusted, but the balances achieved for the interactions are not shown in order to not overload the supplementary material with information.

Table S15. The covariate balance achieved by entropy balancing in sex, age group and SES for the model of 2 or more diagnoses.

**Before weighting**	**With 2 or more diagnoses**			**Without mental disorder**		
	Mean	Variance	Skewness	Mean	Variance	Skewness
Sex[a]	0,5400	0,2484	-0,1607	0,5088	0,2499	-0,0354
Age group[b]	2,0630	0,8965	-0,6511	1,0840	1,3420	0,5373
SES[c]	0,9268	0,3493	0,0189	0,6401	0,3745	0,3973
**After weighting**	**With 2 or more diagnoses**			**Without mental disorder**		
	Mean	Variance	Skewness	Mean	Variance	Skewness
Sex[a]	0,5400	0,2484	-0,1607	0,5400	0,2484	-0,1606
Age group[b]	2,0630	0,8965	-0,6511	2,0630	0,8965	-0,6510
SES[c]	0,9268	0,3493	0,0189	0,9268	0,3493	0,0189

[a] Sex: 0=women; 1=men.

[b] Age group: 0=0-12 years; 1=13-18 years; 2=18-24 years; 3=25-30 years.

[c] SES: 0=high; 1=medium; 2=low.

* Comoments of the joint distribution of the covariates were also adjusted, but the balances achieved for the interactions are not shown in order to not overload the supplementary material with information.

Table S16. Two-part model parameters for any mental disorder.

Part 1	β	Standard error	exp(β)	Low CI	High CI	p-value[a]
Any mental disorder: yes	1.42	0.02	4.13	3.99	4.27	<0.001
Sex: male	-0.35	0.01	0.71	0.69	0.72	<0.001
Age group: 13-18	-0.31	0.02	0.74	0.71	0.76	<0.001
Age group: 19-25	-0.41	0.02	0.66	0.64	0.68	<0.001
Age group: 26-30	-0.49	0.02	0.62	0.60	0.64	<0.001
SES: medium	-0.05	0.01	0.95	0.93	0.97	<0.001
SES: low	0.10	0.02	1.11	1.06	1.16	<0.001
(Intercept)	2.35	0.02	10.52	10.18	10.87	<0.001
**Part 2**	**β**	**Standard error**	**exp(β)**	**Low CI**	**High CI**	**p-value[b]**
Any mental disorder: yes	0.81	0.01	2.25	2.20	2.30	<0.001
Sex: male	-0.18	0.01	0.83	0.81	0.85	<0.001
Age group: 13-18	-0.03	0.02	0.97	0.94	1.00	0.057
Age group: 19-25	-0.07	0.02	0.94	0.91	0.97	<0.001
Age group: 26-30	0.08	0.02	1.09	1.06	1.12	<0.001
SES: medium	0.12	0.01	1.12	1.10	1.15	<0.001
SES: low	0.50	0.03	1.65	1.56	1.74	<0.001
(Intercept)	5.73	0.02	307.56	298.53	316.87	<0.001

[a] Calculated using a logit model.

[b] Calculated using general linear models (family: gamma, link: log).

Table S17. Two-part model parameters for substance use.

**Part 1**	**β**	**Standard error**	**exp(β)**	**Low CI**	**High CI**	**p-value[a]**
Substance use: yes	1.58	0.04	4.86	4.52	5.23	<0.001
Sex: male	-0.48	0.02	0.62	0.59	0.64	<0.001
Age group: 13-18	-0.40	0.06	0.67	0.59	0.76	<0.001
Age group: 19-25	-0.59	0.06	0.55	0.49	0.62	<0.001
Age group: 26-30	-0.70	0.06	0.50	0.44	0.56	<0.001
SES: medium	-0.10	0.02	0.91	0.86	0.95	<0.001
SES: low	0.08	0.04	1.08	1.00	1.17	0.060
(Intercept)	2.64	0.06	14.00	12.44	15.75	<0.001
**Part 2**	**β**	**Standard error**	**exp(β)**	**Low CI**	**High CI**	**p-value[b]**
Substance use: yes	1.14	0.03	3.11	2.96	3.28	<0.001
Sex: male	-0.20	0.03	0.82	0.78	0.87	<0.001
Age group: 13-18	-0.07	0.22	0.93	0.60	1.44	0.756
Age group: 19-25	-0.23	0.22	0.79	0.52	1.21	0.286
Age group: 26-30	-0.18	0.21	0.84	0.55	1.28	0.411
SES: medium	0.20	0.03	1.22	1.14	1.30	<0.001
SES: low	0.94	0.07	2.55	2.23	2.93	<0.001
(Intercept)	5.83	0.22	340.09	222.88	518.94	<0.001

[a] Calculated using a logit model.

[b] Calculated using general linear models (family: gamma, link: log).

Table S18. Two-part model parameters for anxiety.

Part 1	β	Standard error	exp(β)	Low CI	High CI	p-value[a]
Anxiety: yes	1.68	0.03	5.34	5.05	5.65	<0.001
Sex: male	-0.40	0.01	0.67	0.65	0.69	<0.001
Age group: 13-18	-0.29	0.04	0.75	0.69	0.80	<0.001
Age group: 19-25	-0.31	0.04	0.74	0.69	0.79	<0.001
Age group: 26-30	-0.40	0.03	0.67	0.62	0.72	<0.001
SES: medium	-0.09	0.02	0.91	0.88	0.95	<0.001
SES: low	0.07	0.03	1.07	1.01	1.14	0.020
(Intercept)	2.32	0.03	10.14	9.48	10.84	<0.001
**Part 2**	**β**	**Standard error**	**exp(β)**	**Low CI**	**High CI**	**p-value[b]**
Anxiety: yes	0.90	0.02	2.46	2.38	2.54	<0.001
Sex: male	-0.21	0.02	0.81	0.78	0.84	<0.001
Age group: 13-18	0.14	0.03	1.15	1.07	1.23	<0.001
Age group: 19-25	0.01	0.03	1.01	0.96	1.06	0.694
Age group: 26-30	0.14	0.02	1.15	1.10	1.20	<0.001
SES: medium	0.12	0.02	1.13	1.09	1.18	<0.001
SES: low	0.57	0.04	1.77	1.62	1.93	<0.001
(Intercept)	5.66	0.03	286.57	271.70	302.25	<0.001

[a] Calculated using a logit model.

[b] Calculated using general linear models (family: gamma, link: log).

Table S19. Two-part model parameters for mood disorders.

**Part 1**	**β**	**Standard error**	**exp(β)**	**Low CI**	**High CI**	**p-value**[a]
Mood disorders: yes	1.95	0.07	7.05	6.13	8.09	<0.001
Sex: male	-0.39	0.03	0.68	0.64	0.71	<0.001
Age group: 13-18	-0.34	0.05	0.71	0.64	0.79	<0.001
Age group: 19-25	-0.47	0.05	0.62	0.57	0.69	<0.001
Age group: 26-30	-0.54	0.05	0.58	0.53	0.64	<0.001
SES: medium	-0.04	0.03	0.96	0.91	1.03	0.254
SES: low	0.11	0.04	1.12	1.03	1.22	0.012
(Intercept)	2.41	0.05	11.10	10.04	12.27	<0.001
**Part 2**	**β**	**Standard error**	**exp(β)**	**Low CI**	**High CI**	**p-value**[b]
Mood disorders: yes	1.69	0.03	5.43	5.09	5.79	<0.001
Sex: male	-0.08	0.04	0.93	0.86	1.00	0.038
Age group: 13-18	0.32	0.06	1.37	1.22	1.55	<0.001
Age group: 19-25	0.16	0.05	1.17	1.07	1.28	0.001
Age group: 26-30	0.27	0.04	1.32	1.21	1.43	<0.001
SES: medium	0.09	0.05	1.09	1.00	1.19	0.048
SES: low	0.60	0.07	1.83	1.59	2.11	<0.001
(Intercept)	5.51	0.05	246.60	224.41	270.98	<0.001

[a] Calculated using a logit model.

[b] Calculated using general linear models (family: gamma, link: log).

Table S20. Two-part model parameters for psychosis and personality disorders.

**Part 1**	**β**	**Standard error**	**exp(β)**	**Low CI**	**High CI**	**p-value[a]**
Psychosis and personality disorders: yes	1.84	0.08	6.28	5.36	7.36	<0.001
Sex: male	-0.37	0.03	0.69	0.65	0.73	<0.001
Age group: 13-18	-0.36	0.06	0.70	0.62	0.78	<0.001
Age group: 19-25	-0.51	0.06	0.60	0.54	0.67	<0.001
Age group: 26-30	-0.60	0.05	0.55	0.49	0.61	<0.001
SES: medium	-0.11	0.04	0.90	0.83	0.97	0.004
SES: low	0.03	0.05	1.03	0.94	1.14	0.509
(Intercept)	2.49	0.06	12.04	10.78	13.46	<0.001
**Part 2**	**β**	**Standard error**	**exp(β)**	**Low CI**	**High CI**	**p-value[b]**
Psychosis and personality disorders: yes	1.94	0.04	6.94	6.39	7.54	<0.001
Sex: male	-0.17	0.05	0.85	0.77	0.93	0.001
Age group: 13-18	0.23	0.07	1.25	1.10	1.43	0.001
Age group: 19-25	0.22	0.06	1.25	1.11	1.40	<0.001
Age group: 26-30	0.29	0.05	1.34	1.21	1.49	<0.001
SES: medium	0.11	0.06	1.12	1.00	1.25	0.042
SES: low	0.60	0.08	1.82	1.55	2.13	<0.001
(Intercept)	5.51	0.06	247.10	219.76	277.83	<0.001

[a] Calculated using a logit model.

[b] Calculated using general linear models (family: gamma, link: log).

Table S21. Two-part model parameters for ADHD.

**Part 1**	**β**	**Standard error**	**exp(β)**	**Low CI**	**High CI**	**p-value**[a]
ADHD: yes	1.32	0.04	3.75	3.48	4.04	<0.001
Sex: male	-0.19	0.02	0.83	0.79	0.87	<0.001
Age group: 13-18	-0.35	0.03	0.70	0.66	0.75	<0.001
Age group: 19-25	-0.59	0.03	0.55	0.52	0.59	<0.001
Age group: 26-30	-0.74	0.04	0.48	0.44	0.52	<0.001
SES: medium	0.04	0.02	1.04	0.99	1.09	0.118
SES: low	0.15	0.04	1.16	1.08	1.25	<0.001
(Intercept)	2.31	0.03	10.07	9.42	10.77	<0.001
**Part 2**	**β**	**Standard error**	**exp(β)**	**Low CI**	**High CI**	**p-value**[b]
ADHD: yes	0.80	0.03	2.22	2.09	2.35	<0.001
Sex: male	-0.12	0.04	0.89	0.82	0.96	0.004
Age group: 13-18	-0.06	0.03	0.94	0.89	1.00	0.041
Age group: 19-25	-0.14	0.04	0.87	0.81	0.94	<0.001
Age group: 26-30	0.01	0.05	1.01	0.91	1.12	0.804
SES: medium	0.08	0.04	1.09	1.01	1.17	0.022
SES: low	0.34	0.05	1.41	1.27	1.56	<0.001
(Intercept)	5.74	0.04	311.68	286.88	338.61	<0.001

[a] Calculated using a logit model.

[b] Calculated using general linear models (family: gamma, link: log).

Table S22. Two-part model parameters for conduct disorders.

**Part 1**	**β**	**Standard error**	**exp(β)**	**Low CI**	**High CI**	**p-value[a]**
Conduct disorders: yes	1.29	0.03	3.63	3.41	3.86	<0.001
Sex: male	-0.22	0.02	0.81	0.78	0.83	<0.001
Age group: 13-18	-0.29	0.02	0.75	0.72	0.79	<0.001
Age group: 19-25	-0.41	0.03	0.66	0.63	0.69	<0.001
Age group: 26-30	-0.49	0.03	0.61	0.58	0.65	<0.001
SES: medium	-0.04	0.02	0.96	0.92	1.00	0.028
SES: low	0.12	0.03	1.12	1.06	1.19	<0.001
(Intercept)	2.27	0.02	9.72	9.30	10.16	<0.001
**Part 2**	**β**	**Standard error**	**exp(β)**	**Low CI**	**High CI**	**p-value[b]**
Conduct disorders: yes	0.93	0.02	2.52	2.41	2.64	<0.001
Sex: male	-0.08	0.03	0.93	0.88	0.98	0.004
Age group: 13-18	0.06	0.03	1.07	1.01	1.12	0.012
Age group: 19-25	0.07	0.04	1.07	1.00	1.15	0.050
Age group: 26-30	0.30	0.04	1.35	1.24	1.46	<0.001
SES: medium	0.08	0.03	1.08	1.03	1.14	0.003
SES: low	0.42	0.05	1.52	1.37	1.68	<0.001
(Intercept)	5.62	0.03	275.55	260.62	291.33	<0.001

[a] Calculated using a logit model.

[b] Calculated using general linear models (family: gamma, link: log).

Table S23. Two-part model parameters for eating disorders.

**Part 1**	**β**	**Standard error**	**exp(β)**	**Low CI**	**High CI**	**p-value[a]**
Eating disorders: yes	1.43	0.08	4.18	3.57	4.89	<0.001
Sex: male	-0.28	0.05	0.75	0.69	0.83	<0.001
Age group: 13-18	-0.23	0.06	0.80	0.70	0.91	<0.001
Age group: 19-25	-0.27	0.07	0.76	0.67	0.88	<0.001
Age group: 26-30	-0.27	0.07	0.76	0.67	0.87	<0.001
SES: medium	-0.04	0.04	0.96	0.88	1.05	0.353
SES: low	0.18	0.07	1.20	1.05	1.37	0.008
(Intercept)	2.20	0.06	8.99	8.03	10.07	<0.001
**Part 2**	**β**	**Standard error**	**exp(β)**	**Low CI**	**High CI**	**p-value[b]**
Eating disorders: yes	1.13	0.05	3.10	2.79	3.44	<0.001
Sex: male	-0.25	0.06	0.78	0.70	0.88	<0.001
Age group: 13-18	0.22	0.07	1.25	1.09	1.43	0.001
Age group: 19-25	0.01	0.07	1.01	0.89	1.15	0.870
Age group: 26-30	0.25	0.09	1.28	1.08	1.51	0.004
SES: medium	0.13	0.06	1.14	1.02	1.28	0.022
SES: low	0.60	0.13	1.83	1.42	2.35	<0.001
(Intercept)	5.64	0.05	280.49	253.92	309.83	<0.001

[a] Calculated using a logit model.

[b] Calculated using general linear models (family: gamma, link: log).

Table S24. Two-part model parameters for self-harm.

**Part 1**	**β**	**Standard error**	**exp(β)**	**Low CI**	**High CI**	**p-value[a]**
Self-harm: yes	2.42	0.32	11.25	6.02	21.01	<0.001
Sex: male	-0.19	0.06	0.83	0.73	0.93	0.002
Age group: 13-18	-0.59	0.08	0.56	0.48	0.65	<0.001
Age group: 19-25	-0.67	0.11	0.51	0.41	0.63	<0.001
Age group: 26-30	-0.81	0.09	0.44	0.38	0.53	<0.001
SES: medium	0.17	0.13	1.19	0.92	1.54	0.187
SES: low	0.32	0.14	1.38	1.06	1.81	0.019
(Intercept)	2.35	0.06	10.44	9.26	11.77	<0.001
**Part 2**	**β**	**Standard error**	**exp(β)**	**Low CI**	**High CI**	**p-value[b]**
Self-harm: yes	2.46	0.09	11.71	9.86	13.91	<0.001
Sex: male	-0.26	0.10	0.77	0.64	0.93	0.008
Age group: 13-18	0.90	0.15	2.46	1.85	3.28	<0.001
Age group: 19-25	0.52	0.11	1.68	1.34	2.10	<0.001
Age group: 26-30	0.90	0.12	2.47	1.94	3.13	<0.001
SES: medium	-0.30	0.16	0.74	0.54	1.01	0.058
SES: low	0.37	0.20	1.45	0.97	2.15	0.067
(Intercept)	5.35	0.12	210.00	166.06	265.56	<0.001

[a] Calculated using a logit model.

[b] Calculated using general linear models (family: gamma, link: log).

Table S25. Two-part model parameters for 2 or more diagnoses.

**Part 1**	**β**	**Standard error**	**exp(β)**	**Low CI**	**High CI**	**p-value[a]**
2 or more diagnoses: yes	1.99	0.05	7.33	6.68	8.04	<0.001
Sex: male	-0.41	0.02	0.66	0.64	0.69	<0.001
Age group: 13-18	-0.35	0.04	0.71	0.65	0.76	<0.001
Age group: 19-25	-0.49	0.04	0.61	0.57	0.66	<0.001
Age group: 26-30	-0.58	0.04	0.56	0.52	0.60	<0.001
SES: medium	-0.07	0.02	0.93	0.89	0.97	0.001
SES: low	0.08	0.03	1.09	1.02	1.16	0.012
(Intercept)	2.47	0.04	11.80	10.93	12.73	<0.001
**Part 2**	**β**	**Standard error**	**exp(β)**	**Low CI**	**High CI**	**p-value[b]**
2 or more diagnoses: yes	1.41	0.02	4.08	3.89	4.28	<0.001
Sex: male	-0.16	0.03	0.86	0.81	0.90	<0.001
Age group: 13-18	0.11	0.04	1.12	1.03	1.22	0.005
Age group: 19-25	-0.01	0.04	0.99	0.92	1.06	0.765
Age group: 26-30	0.11	0.04	1.12	1.05	1.20	0.001
SES: medium	0.08	0.03	1.08	1.01	1.15	0.017
SES: low	0.59	0.05	1.80	1.61	2.00	<0.001
(Intercept)	5.67	0.04	290.25	269.22	312.93	<0.001

[a] Calculated using a logit model.

[b] Calculated using general linear models (family: gamma, link: log).

Table S26. Mean and excess cost per patient in € of direct healthcare costs for any mental disorder disaggregated by sex, age group and socioeconomic status.

		Mean cost (€)					
		**Without mental disorder**	**With any mental disorder**	**Excess cost (€)**	**Low CI**	**High CI**	**p-value[a]**
**Sex**	**Female**	310.22	769.75	459.52	443.07	475.97	<0.001
	**Male**	246.24	633.17	386.92	372.27	401.57	<0.001
**Age group**	**1-12**	287.23	702.10	414.88	400.08	429.67	<0.001
	**13-18**	268.77	674.79	406.02	388.69	423.36	<0.001
	**19-24**	255.41	648.38	392.97	376.70	409.24	<0.001
	**25-30**	293.44	750.78	457.34	439.52	475.16	<0.001
**SES**	**High**	242.04	609.80	367.76	353.86	381.67	<0.001
	**Medium**	270.07	684.16	414.09	399.92	428.26	<0.001
	**Low**	403.64	1,006.93	603.29	564.32	642.26	<0.001

[a] Calculated using two-part models.

Table S27. Mean and excess cost per patient in € of direct healthcare costs for substance use disaggregated by sex, age group and socioeconomic status.

		Mean cost (€)					
		**Without mental disorder**	**With substance use**	**Excess cost (€)**	**Low CI**	**High CI**	**p-value[a]**
**Sex**	**Female**	323.38	1,118.38	795.00	738.05	851.95	<0.001
	**Male**	246.85	903.80	656.96	605.38	708.53	<0.001
**Age group**	**1-12**	364.38	1,222.26	857.88	494.90	1,220.86	<0.001
	**13-18**	325.62	1,129.76	804.13	702.14	906.13	<0.001
	**19-24**	269.83	955.06	685.22	625.61	744.83	<0.001
	**25-30**	280.04	1,004.11	724.07	672.46	775.68	<0.001
**SES**	**High**	215.36	762.23	546.87	501.06	592.67	<0.001
	**Medium**	257.97	923.82	665.86	619.54	712.18	<0.001
	**Low**	556.29	1,951.67	1,395.38	1,197.45	1,593.30	<0.001

[a] Calculated using two-part models.

Table S28. Mean and excess cost per patient in € of direct healthcare costs for anxiety disaggregated by sex, age group and socioeconomic status.

		**Mean cost (€)**					
		**Without mental disorder**	**With anxiety**	**Excess cost (€)**	**Low CI**	**High CI**	**p-value[a]**
**Sex**	**Female**	320.56	880.24	559.68	532.29	587.07	<0.001
	**Male**	244.58	703.99	459.41	432.04	486.78	<0.001
**Age group**	**1-12**	276.06	744.95	468.89	442.48	495.30	<0.001
	**13-18**	305.62	848.36	542.75	501.29	584.20	<0.001
	**19-24**	268.63	746.72	478.08	450.77	505.40	<0.001
	**25-30**	301.35	847.04	545.69	518.05	573.33	<0.001
**SES**	**High**	250.76	695.96	445.20	419.14	471.25	<0.001
	**Medium**	279.84	784.59	504.75	480.67	528.83	<0.001
	**Low**	448.46	1,235.47	787.02	711.94	862.09	<0.001

[a] Calculated using two-part models.

Table S29. Mean and excess cost per patient in € of direct healthcare costs for mood disorders disaggregated by sex, age group and socioeconomic status.

		Mean cost (€)					
		**Without mental disorder**	**With mood disorders**	**Excess cost (€)**	**Low CI**	**High CI**	**p-value[a]**
**Sex**	**Female**	317.33	1,923.03	1,605.70	1,467.36	1,744.05	<0.001
	**Male**	277.40	1,762.57	1,485.16	1,354.55	1,615.77	<0.001
**Age group**	**1-12**	251.14	1,491.51	1,240.37	1,125.97	1,354.78	<0.001
	**13-18**	331.65	2,036.48	1,704.83	1,480.91	1,928.75	<0.001
	**19-24**	276.74	1,726.35	1,449.61	1,313.09	1,586.14	<0.001
	**25-30**	308.48	1,940.42	1,631.95	1,485.49	1,778.40	<0.001
**SES**	**High**	250.82	1,556.59	1,305.77	1,165.87	1,445.68	<0.001
	**Medium**	272.77	1,700.33	1,427.56	1,317.80	1,537.32	<0.001
	**Low**	466.16	2,855.34	2,389.18	2,057.72	2,720.64	<0.001

[a] Calculated using two-part models.

Table S30. Mean and excess cost per patient in € of direct healthcare costs for psychosis and personality disorders disaggregated by sex, age group and socioeconomic status.

		**Mean cost (€)**					
		**Without mental disorder**	**With psychosis and personality disorders**	**Excess cost (€)**	**Low CI**	**High CI**	**p-value[a]**
**Sex**	**Female**	333.78	2,584.07	2,250.29	1,994.62	2,505.97	<0.001
	**Male**	267.84	2,166.75	1,898.91	1,708.13	2,089.69	<0.001
**Age group**	**1-12**	246.66	1,869.28	1,622.62	1,437.03	1,808.20	<0.001
	**13-18**	296.26	2,324.01	2,027.76	1,727.22	2,328.30	<0.001
	**19-24**	288.65	2,305.63	2,016.98	1,763.92	2,270.04	<0.001
	**25-30**	305.68	2,471.82	2,166.14	1,937.03	2,395.24	<0.001
**SES**	**High**	237.44	1,875.11	1,637.67	1,425.41	1,849.92	<0.001
	**Medium**	261.98	2,096.61	1,834.63	1,660.37	2,008.89	<0.001
	**Low**	433.61	3,410.98	2,977.37	2,528.58	3,426.17	<0.001

[a] Calculated using two-part models.

Table S31. Mean and excess cost per patient in € of direct healthcare costs for ADHD disaggregated by sex, age group and socioeconomic status.

		Mean cost (€)		Excess cost (€)	Low CI	High CI	p-value[a]
		Without mental disorder	With ADHD				
**Sex**	**Female**	277.05	677.06	400.01	349.79	450.23	<0.001
	**Male**	240.22	597.91	357.69	322.38	393.00	<0.001
**Age group**	**1-12**	280.91	671.19	390.28	355.75	424.80	<0.001
	**13-18**	253.46	623.31	369.84	329.34	410.35	<0.001
	**19-24**	226.53	571.10	344.57	302.51	386.63	<0.001
	**25-30**	256.94	659.47	402.54	349.72	455.35	<0.001
**SES**	**High**	228.34	567.55	339.21	299.54	378.89	<0.001
	**Medium**	249.26	617.23	367.97	330.78	405.16	<0.001
	**Low**	327.96	803.38	475.42	414.75	536.09	<0.001

[a] Calculated using two-part models.

Table S32. Mean and excess cost per patient in € of direct healthcare costs for conduct disorders disaggregated by sex, age group and socioeconomic status.

		**Mean cost (€)**					
		**Without mental disorder**	**With conduct disorders**	**Excess cost (€)**	**Low CI**	**High CI**	**p-value[a]**
**Sex**	**Female**	292.41	807.87	515.46	474.63	556.28	<0.001
	**Male**	263.01	741.01	478.00	442.04	513.97	<0.001
**Age group**	**1-12**	261.91	715.74	453.83	425.82	481.83	<0.001
	**13-18**	269.72	755.06	485.34	444.24	526.45	<0.001
	**19-24**	266.94	756.73	489.79	438.86	540.71	<0.001
	**25-30**	331.51	947.11	615.60	553.66	677.54	<0.001
**SES**	**High**	245.88	687.06	441.18	405.20	477.16	<0.001
	**Medium**	265.11	743.88	478.77	445.27	512.27	<0.001
	**Low**	378.36	1,046.53	668.16	588.44	747.89	<0.001

[a] Calculated using two-part models.

Table S33. Mean and excess cost per patient in € of direct healthcare costs for eating disorders disaggregated by sex, age group and socioeconomic status.

		Mean cost (€)					
		**Without mental disorder**	**With eating disorders**	**Excess cost (€)**	**Low CI**	**High CI**	**p-value[a]**
**Sex**	**Female**	325.71	1,110.67	784.96	659.74	910.18	<0.001
	**Male**	244.99	859.21	614.23	512.44	716.01	<0.001
**Age group**	**1-12**	272.30	917.82	645.51	543.43	747.60	<0.001
	**13-18**	330.66	1,136.64	805.98	668.41	943.56	<0.001
	**19-24**	266.54	919.91	653.37	540.72	766.03	<0.001
	**25-30**	337.26	1,164.20	826.95	651.22	1,002.68	<0.001
**SES**	**High**	260.41	894.16	633.74	530.97	736.51	<0.001
	**Medium**	295.35	1,018.13	722.77	609.79	835.76	<0.001
	**Low**	485.81	1,642.13	1,156.32	817.79	1,494.85	<0.001

[a] Calculated using two-part models.

Table S34. Mean and excess cost per patient in € of direct healthcare costs for self-harm disaggregated by sex, age group and socioeconomic status.

		Mean cost (€)		Excess cost (€)	Low CI	High CI	p-value[a]
		Without mental disorder	With self-harm				
Sex	Female	353.23	4,727.23	4,374.00	3,394.02	5,353.99	<0.001
	Male	264.87	3,636.31	3,371.44	2,688.57	4,054.30	<0.001
Age group	1-12	163.15	2,057.81	1,894.66	1,384.56	2,404.75	<0.001
	13-18	378.31	5,039.74	4,661.43	3,444.02	5,878.84	<0.001
	19-24	254.69	3,429.39	3,174.70	2,359.78	3,989.62	<0.001
	25-30	366.42	5,027.00	4,660.58	3,672.14	5,649.01	<0.001
SES	High	338.81	4,691.77	4,352.96	2,877.91	5,828.02	<0.001
	Medium	257.95	3,485.80	3,227.84	2,652.39	3,803.29	<0.001
	Low	513.80	6,816.02	6,302.22	4,250.01	8,354.43	<0.001

[a] Calculated using two-part models.

Table S35. Mean and excess cost per patient in € of direct healthcare costs for 2 or more diagnoses disaggregated by sex, age group and socioeconomic status.

		Mean cost (€)		Excess cost (€)	Low CI	High CI	p-value[a]
		**Without mental disorder**	**With 2 or more diagnoses**				
**Sex**	**Female**	316.77	1,445.56	1,128.78	1,058.10	1,199.47	<0.001
	**Male**	255.40	1,226.06	970.66	905.35	1,035.97	<0.001
**Age group**	**1-12**	281.12	1,252.96	971.85	900.13	1,043.56	<0.001
	**13-18**	303.00	1,396.84	1,093.83	1,001.45	1,186.22	<0.001
	**19-24**	261.92	1,227.82	965.90	894.79	1,037.01	<0.001
	**25-30**	292.53	1,388.20	1,095.67	1,024.04	1,167.30	<0.001
**SES**	**High**	244.92	1,141.77	896.84	825.56	968.12	<0.001
	**Medium**	261.98	1,232.33	970.35	914.27	1,026.43	<0.001
	**Low**	445.25	2,055.24	1,609.98	1,436.70	1,783.27	<0.001

[a] Calculated using two-part models.
